# Co-Encapsulation of Chlorin e6 and Chemotherapeutic Drugs in a PEGylated Liposome Enhance the Efficacy of Tumor Treatment: Pharmacokinetics and Therapeutic Efficacy

**DOI:** 10.3390/pharmaceutics11110617

**Published:** 2019-11-17

**Authors:** Po-Chun Peng, Ruey-Long Hong, Tsuimin Tsai, Chin-Tin Chen

**Affiliations:** 1 Department of Biochemical Science and Technology, College of Life Science, National Taiwan University, Taipei 10617, Taiwan; swaigod@hotmail.com; 2 Department of Oncology, National Taiwan University Hospital, Taipei 10016, Taiwan; rlhong@ntu.edu.tw; 3 Graduate Institute of Biomedical Materials and Engineering, Graduate School of Dentistry, Taipei Medical University, Taipei 11043, Taiwan; tmtsai00@gmail.com

**Keywords:** liposome, triggered release, chemotherapy, photodynamic therapy

## Abstract

Long-circulating PEG-modified liposome has been shown to improve pharmacokinetic properties and reduce systemic toxicity in cancer treatment. However, drug bioavailability from liposome remains a major challenge to the improvement of its therapeutic efficacy. Previously, we designed a PEGylated dual-effect liposome (named as PL-Dox-Ce6) with chlorin e6 incorporated in the lipid bilayer and Doxorubicin encapsulated in the interior. In this study, another dual-effect liposome with cisplatin encapsulated in the interior was further developed. The pharmacokinetics of these two dual-effect liposomes were studied in tumor-bearing mice. Based on the kinetic data of tumor and plasma, light irradiation was applied onto the tumors at different time points after drug administration to compare the therapeutic efficacy. We demonstrated that a single dose of the dual-effect liposomes combined with two doses of light irradiation can completely eradicate over 90% of the tumor in mice alone with significant survival rate and no toxicity. Thus, this study established a platform that utilizes the dual-effect liposome which combines photodynamic therapy and chemotherapy to improve the therapeutic outcomes of tumors.

## 1. Introduction

Chemotherapeutic drugs, such as Doxorubicin (Dox) and Cisplatin (cDDP), are highly effective anticancer agents approved for the treatment of a variety of human cancers. However, severe dose-limiting side effects might limit their clinical use [1,2]. Liposomal drug delivery systems have been studied extensively to increase the therapeutic efficacy of chemotherapy and several formulations have been in clinical use [3,4]. A significant step in the development of long-circulating liposomes came with the inclusion of synthetic polymer poly(ethylene glycol) (PEG) in liposome composition. PEGylation on the surface of liposome has been shown to reduce mononuclear phagocyte system uptake and extend their blood-circulation time due to the retarding recognition by the reticulo-endothelial system (RES) [5]. These PEGylated liposomes are referred to as sterically stabilized or stealth liposomes. The substantial side-effect of Dox or cDDP was successfully resolved when encapsulating these drugs in the long-circulating stealth liposomes. In addition, these liposomal drugs have demonstrated the alteration of biodistribution, the improvement of pharmacokinetic properties and the reduction of toxicity [4,6,7]. Presently, the PEGylated liposomal doxorubicin (Dox) (Caelyx^®^ in Europe and Doxil^®^ in the USA) and liposomal irinotecan (Onivyde^®^) were approved for clinical use in cancer therapy [4].

Despite these promising aspects, patients receiving PEGylated liposomes had a greater chance of experiencing hand-foot syndrome due to the long-circulation of the liposomes in the blood circulation [8]. In addition, stealth liposomes impede their therapeutic efficacy due to the slow and passive drug release from these liposomes and the prevention of uptake of PEGylated liposomes by target cells in clinical practice [9]. Therefore, low drug bioavailability from liposomal formulations and limited tumor accumulation remain major challenges to further improve the therapeutic efficacy of liposomal drugs. As a consequence, finding methods to selectively destabilize liposomal drug formulations in the tumor area is a major barrier to the liposome field, which, if overcome, could lead to substantial increases in drug bioavailability at the tumor site and therapeutic efficacy. Several approaches are being studied to improve the bioavailability of liposomal drugs [10,11,12]. These approaches, including hyperthermia, irradiation, and the administration of vasoactive factors, have been explored to further enhance the effective permeability of tumor vessels to macromolecular agents. For example, light-triggered liposome has been developed by introducing photoreactive groups in the phospholipid molecule by chemical synthesis [13,14,15].

Photodynamic therapy (PDT) is a therapeutic modality for cancer treatment. The principle of PDT combines the photosensitizer and light irradiation to generate reactive oxygen species, which can cause tumor cell killing as well as damage to the exposed microvasculature [16,17,18]. Due to the vessel leakage, it has been shown that the combination of PDT and Doxil can led to a significant potentiation in tumor control without concomitant enhancement of systemic or local toxicity [19].

In a previous study, we developed a PEGylated dual-effect liposome (named PL-Dox-Ce6) with chlorin e6 (Ce6) incorporated in the lipid bilayer and Doxorubicin (Dox) encapsulated in the interior [20]. The in vivo study of PL-Dox-Ce6 revealed a significant therapeutic efficacy compared to that of liposomal Ce6 and liposomal Dox alone or in combination, suggesting that this dual-effect PL-Dox-Ce6 could provide clinical advantages in the combination regimen of PDT and chemotherapy. In this study, we further addressed the biodistribution and pharmacokinetics in BALB/c mice bearing C26 colon tumor. The impact of irradiation scheme on therapeutic efficacy of PL-Dox-Ce6 for antitumor therapy was also evaluated to develop a more effective way of using PL-Dox-Ce6. In addition, we developed another PEGylated dual-effect liposome with cisplatin (cDDP) encapsulated in the interior (named as PL-cDDP-Ce6) to verify the advantage of this liposomal platform. Based on the in vivo pharmacokinetic data, optimal time points of light irradiation were introduced to increase the therapeutic efficacy. Finally, we showed that a single dose of dual-effect liposomal drug combined with two doses of light irradiation could completely eradicate 90% of the tumor in C26-bearing mice as well as in H460-bearing mice alone with a significant survival rate and no toxicity. 

## 2. Materials and Methods

### 2.1. Chemicals

1,2-distearoyl-sn-glycero-3-phosphoethanolamine-*N*-[methoxy(polyethyleneglycol)]-2000 (DSPE-PEG2000) and 1,2-distearoyl-sn-glycero-3-phosphocholine (DSPC) were purchased from Avanti Polar Lipids (Birmingham, AL, USA). These phospholipids were dissolved in chloroform, sealed in ampoules and stored at −80 °C before use. Doxorubicin (Dox) and chlorin e6 (Ce6) was obtained from the Taiwan Liposome Company (Taipei, Taiwan) and the Frontier Scientific Corporation (Logan, UT, USA), respectively. Cholesterol and cisplatin (cDDP) were purchased from Sigma-Aldrich (St Louis, MO, USA). 

### 2.2. Cell Cultures 

Human melanoma A375 and lung H460 cancer cells were grown in Dulbecco’s Modified Eagle Medium (DMEM) supplemented with 10% fetal bovine serum (FBS). Mouse colon tumor C26 cells were grown in RPMI1640 medium containing 10% FBS. All the cells were cultured at 37 °C in a humidified atmosphere of 5% CO_2_. 

### 2.3. Preparation and Characterization of Dual-Effect Liposome

The PL-Dox-Ce6 liposomes were prepared according to the method described previously [20]. Briefly, DSPC/DSPE-PEG2000/cholesterol (10:0.2:5 μmole) were dissolved in chloroform, then Ce6 solution in DMF was added to the mixture. Ce6 was incorporated in liposomes by the thin-film hydration method. Liposomes were then extruded through polycarbonate membranes. Size exclusion chromatography was used to remove the untrapped free Ce6 and lipids by using a Sephadex G-50 column. The final dispersion containing PEGylated liposomal Ce6 (PL-Ce6) was suspended in 0.9% (*w/v*) NaCl and stored at 4 °C until further use. The PL-Dox-Ce6 was prepared by loading Dox into the prepared PL-Ce6 by transmembrane amine gradients. Size exclusion chromatography was used to remove the untrapped free-form Dox by using a Sephadex G-50 column. The final dispersion containing liposomes co-encapsulated Ce6 and Dox (PL-Dox-Ce6) was suspended in 0.9% (*w/v*) NaCl and stored at 4 °C. 

The PL-cDDP-Ce6 was prepared by an ethanol injection method. Briefly, cDDP (8 mg/mL) was dissolved in 0.9% (*w/v*) NaCl at 65 °C. Ce6 and Lipids (DSPC/DSPE-PEG2000/cholesterol 10:0.2:5 molar ratio) were dissolved in ethanol. The liposome was formed by adding this ethanolic solution to the cDDP mixture. The final lipid concentration was 150.2 μmole in 10% ethanol at 65 °C. The mixture was kept at sonication for 1 h at 65 °C and then extruded through 100 nm pore size polycarbonate membranes at 65 °C. Size exclusion chromatography was used to remove the untrapped free-form cDDP and Ce6 by using a Sephadex G-50 column. The final dispersion containing liposomes co-encapsulated Ce6 and cDDP (PL-cDDP-Ce6) was suspended in 0.9% (*w/v*) NaCl and stored at 4 °C. 

The characterization of liposome was examined according to the method described previously [20]. The size distribution of liposome was measured with dynamic light scattering using a particle sizer (SZ-100, HORIBA, Kyoto, Japan). Ce6, Dox and cDDP entrapped in the liposome was determined by disrupting the liposome bilayer with absolute ethanol to release the entrapped drugs. UV–visible spectroscopy at λ = 400 and 470 nm was used to estimate the concentration of Ce6 and Dox in the solution, respectively (DU800 Beckman Coulter, Fullerton, CA., USA). The amount of encapsulated cDDP was determined by HPLC based on the method of pre-column derivatization of cDDP with sodium diethyldithiocarbamate (DDTC) by quantitation against a NiCl_2_ internal standard [21]. Briefly, 10 µg NiCl_2_ and 5 mg DDTC in 700 µl of 0.1N NaOH were added into a 300 µL disrupted sample. The mixture was incubated for 1 h at 50 °C and further extracted with chloroform. The chloroform extract containing Pt(DDTC)_2_ and Ni(DDTC)_2_ complexes was separated by HPLC on a C18 column using water and methanol (1:3) as the mobile phase. The complexes were detected at a wavelength of 254 nm with flow rate maintained at 1 mL/min. 

### 2.4. Stability Analysis and In Vitro Drug Release Profile of Dual-Effect Liposome

For analyzing stability, 0.5 mL aliquots of the PL-Dox-Ce6 or PL-cDDP-Ce6 suspension were placed in Eppendorf vials, sealed and stored at 4 °C and kept in the dark. At intervals, liposome suspension from the stored vials was passed through a Sephadex G-50 column to collect the PL-Dox-Ce6 or PL-cDDP-Ce6. After disrupting the collected liposome, the amount of encapsulated Ce6, Dox or cDDP was determined. The percentage of encapsulated drugs in the collected PL-Dox-Ce6 or PL-cDDP-Ce6 was used to estimate the stability of liposome.

Light-triggered release experiments were performed by illuminating the liposomal drugs with a home-made 662 nm diode laser delivered an irradiance of 105 mW/cm^2^ and a fluence of 10 J/cm^2^. Drug release from liposomes was studied using a dialysis method in PBS at 37 °C. At the time indicated, the released Dox or cDDP was determined. The released ratio was assessed by measuring the release before and after treatment.

In the stability and release study, the amount of Ce6 and Dox was determined by measuring the fluorescence intensity with a spectrofluorometer (FluoroMax1-4, Horiba Jobin Yvon, NJ, USA). The fluorescence intensity of Ce6 and Dox was determined at a wavelength of excitation/emission of 650/665 nm and 470/586 nm, respectively. cDDP was determined as described in Section 2.3. 

### 2.5. Animal Experiments in Mice

All the procedures conducted with mice were approved by the National Taiwan University Institutional Animal Care and Use Committee (IACUC) (NTU-100-EL-9, 05/2011). Female BALB/c mice (6–8 weeks of age) and nu/nu nude mice (6–8 weeks of age) were purchased from the National Laboratory Animal Center and BioLASCO Taiwan, respectively (Taipei, Taiwan). Mouse colon tumor C26 cells with dimensions of 2 × 10^5^ were injected into the right back of BALB/c mice by subcutaneous injection. H460 human lung cancer cells with dimensions of 1 × 10^6^ were injected into the right back of nu/nu nude mice by subcutaneous injection. When the tumor volumes reached the desired size, mice were grouped for the following studies. 

#### 2.5.1. Biodistribution and Pharmacokinetic Studies of Pl-Dox-Ce6 & Pl-Cddp-Ce6 in C26 Tumor-Bearing Balb/c Mice

BALB/c mice bearing C26 syngeneic tumor (~100 mm^3^) were intravenously injected a single dose of PL-Dox-Ce6 (Ce6: 1.25 mg/kg and Dox: 5.87 mg/kg) or PL-cDDP-Ce6 (Ce6: 1.75 mg/kg and cDDP: 3.54 mg/kg). Eight mice were randomly selected as the control group. At the indicated time points, three mice in each group were anaesthetized and sacrificed. Blood samples were collected through submaxillary punctures and plasma samples were prepared. After perfusion, tumors and mouse organs (liver, heart, spleen, lung and kidney) were removed and homogenized. To the tissue samples was added a 4-folds volume of acidic ethanol (95% ethanol and 0.6 N HCl) and incubated at 4 °C overnight. The supernatant of the samples was collected and centrifuged at 12,000 rpm for 15 min for the following analysis. The relative amounts of Ce6 and Dox were determined by a fluorescence analysis in a spectrofluorometer. The fluorescence intensity of Ce6 and Dox was measured at a wavelength of excitation/emission of 640/653 nm and 470/586 nm, respectively. cDDP in homogenized tissues was determined as described in Section 2.3. The concentrations of Dox, cDDP and Ce6 in tissues were expressed as microequivalents per milliliter of plasma or per gram of tissue.

#### 2.5.2. Tumor Drug Deposition Determination

The total tumor Dox and Dox in the nuclei was quantified according to the methods of Mayer et al. [22]. Briefly, 2 h after liposomes injection, tumors were illuminated with a home-made 662 nm diode laser delivered at an irradiance of 105 mW/cm^2^ and a fluence of 100 J/cm^2^. After light irradiation, tumor tissues were removed at the time indicated and homogenated in lysis buffer [0.25 mol/L sucrose, 5 mmol/L Tris HCl, 1 mmol/L MgSO_4_,1 mmol/L CaCl_2_ (pH 7.6)]. Tumor cell nuclei were isolated from total tumor homogenates by differential centrifugation through a 1.8 mol/L sucrose gradient at 1000 × *g* for 10 min. The total tumor or nuclear Dox was extracted from homogenate by acidic ethanol (95% ethanol and 0.6 N HCl) at 4 °C overnight. The concentrations of Dox were analyzed by fluorescence intensity as described above.

#### 2.5.3. In Vivo Therapeutic Experiments

C26 or H460 tumor-bearing mice were intravenously injected a single dose of liposomal drugs (diluted with 0.9% NaCl) as indicated through the tail vein when the tumor grew up to 100 mm^3^, 300 mm^3^ or 500 mm^3^ in volume. The control group was given 0.9% NaCl. Mice were incubated in the dark before light irradiation. At the time indicated, tumors were irradiated locally with a home-made 662 nm diode laser delivered at an irradiance of 105 mW/cm^2^ and a fluence of 100 J/cm^2^. After the treatment, the tumor size, body weight and survival of the mice were monitored every three days. The end point of the experiment was when the subsequent tumor size reached approximately 2500 mm^3^ in volume, then the animals were killed by CO_2_ inhalation.

### 2.6. Statistics

Data values are represented as mean ± standard deviation (S.D.). Two-way ANOVA was used to analyze the differences in tumor volume and body weight data. A Kaplan–Meier survival curve was used to evaluate the survival rate and the difference was analyzed by a log-rank test. The concentration data of Ce6, Dox and cDDP were analyzed using Student’s *t*-test. A statistical analysis was conducted using IBM SPSS Statistics software, version 17.0. *P*-values < 0.05 were regarded statistically significant for all the analyses.

## 3. Results

### 3.1. Biodistribution and Therapeutic Efficacy of PL-Dox-Ce6

**Figure 1:**
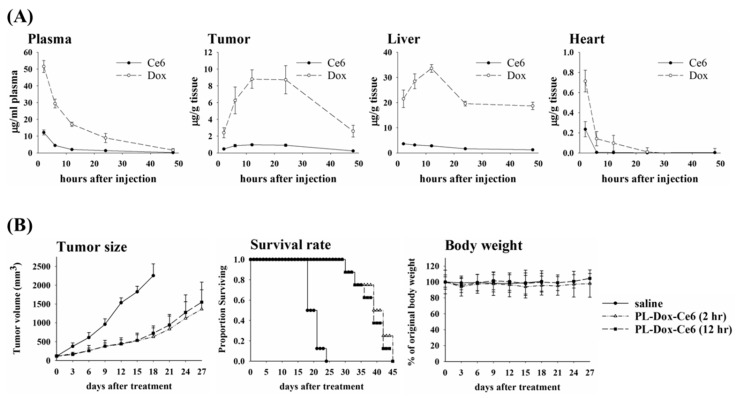
Biodistribution and therapeutic efficacy in BALB/c mice bearing C26 tumor after intravenous injection of PL-Dox-Ce6. (**A**) Time course of Ce6 and Dox concentrations in plasma, tumor, liver and heart after injection of PL-Dox-Ce6 (Ce6: 1.25 mg/kg and Dox: 5.87 mg/kg). Data are expressed as the mean ± standard deviation (S.D.) for each group (*N* = 3). (**B**) Therapeutical efficacy of PL-Dox-Ce6. After liposomal drug (Ce6: 1.75 mg/kg and Dox: 8.3 mg/kg) injection, light irradiation was applied onto the tumor at 2 h and 12 h, respectively. Left panel, tumor size; Middle panel, survival rate; Right panel, body weight. The results are represented as the mean ± S.D. for each group (*N* = 8).

Previously, we have shown that PL-Dox-Ce6 exerted a significant therapeutic efficacy compared to that of liposomal Ce6 (PL-Ce6) and liposomal Dox (PL-Dox) alone or in combination [20]. To address the fate of PL-Dox-Ce6 liposome after administration, the Ce6 and Dox concentrations in plasma and several tissues were determined at different time-points after a single intravenous injection of PL-Dox-Ce6. Figure 1A shows the tissue distribution of PL-Dox-Ce6 as a function of time after injection into a syngeneic C26 tumor-bearing BALB/c mice and the data are summarized in Table S1, Supplementary Materials. As expected, the liposomal drug preferentially accumulates in tissues containing a discontinuous microvasculature, such as tumors, or in organs containing the macrophages of the reticuloendothelial system (RES), such as liver and kidney, while much less were found in non-RES tissues measured (skin, muscle, heart and lung). The plasma levels of Dox and Ce6 were initially high 2 h after injection (averaged 51.57 μg/mL and 12.2μg/mL, respectively) and appeared to decline exponentially, as would normally be expected for an intravenous injection. In contrast, the levels of Dox and Ce6 in tumor reached a peak (averaged 8.81 and 0.97 μg/g, respectively) at 12 h and then maintained as a plateau until 24 h after drug administration. Fourty-eight hours after injection, both Dox and Ce6 were almost undetectable in plasma. 

Our previous study demonstrated that PL-Dox-Ce6 provides a significant improvement in the therapeutic efficacy 2 h after drug administration [20]. However, the present pharmacokinetic (PK) profile indicates that the concentration of Dox and Ce6 reached the plateau between 12 to 24 h in tumor after PL-Dox-Ce6 injection. Therefore, to further evaluate the therapeutic potential of PL-Dox-Ce6, light irradiation at different time points was selected based on the kinetic profiles of PL-Dox-Ce6 in the tumor. A single dose of PL-Dox-Ce6 was administered into the BALB/c mice with C26 tumor of 100 mm^3^ and they received light irradiation either at 2 h or 12 h after drug injection, respectively. Compared with the control group, the mean tumor sizes were significantly reduced in the PL-Dox-Ce6 treated groups (irradiation at either 2 h or 12 h), which is consistent with the increased survival rate (Figure 1B). Compared to the drug contents at 2 h post injection, the levels of Dox (8.81 μg/g) and Ce6 (0.97 μg/g) in tumor are about four-fold and two-fold higher at 12 h, respectively (Table S1, Supplementary Materials). Therefore, a better therapeutic efficacy was reasonably expected to be found in the group that received light irradiation at 12 h post drug administration. To our surprise, there was no significant difference in tumor suppression and survival rate between the groups that received light irradiation either at 2 h or 12 h, as shown in Figure 1B. 

### 3.2. The Impact of the Irradiation Scheme on Drug Accumulation in Tumor

**Figure 2:**
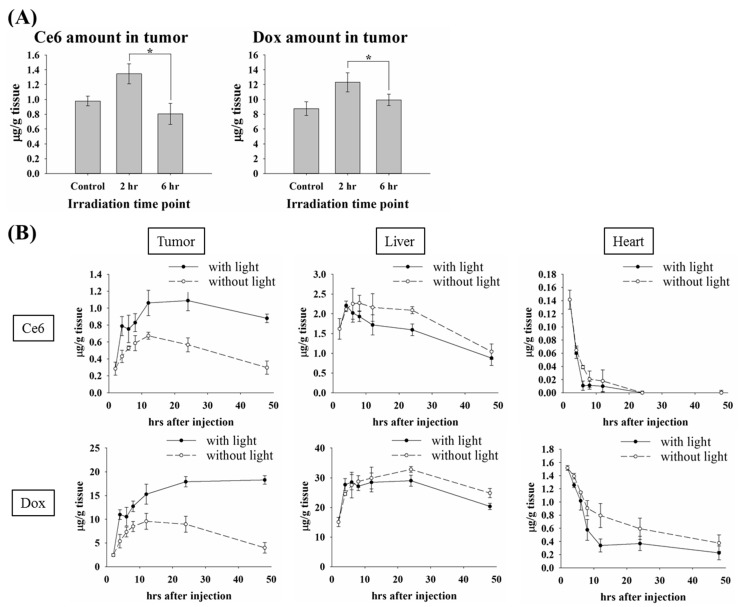
Drug concentrations of Ce6 and Dox in tumor and different tissues of C26 tumor-bearing mice with or without light irradiation. (**A**) Total Dox (right panel) and Ce6 (left panel) levels in whole tumor 12 h after PL-Dox-Ce6 (Ce6: 1.25 mg/kg and Dox: 5.87 mg/kg) administration. After drug administration, tumor tissues were irradiated without (control) or with light (100 J/cm^2^) at 2 h and 6 h, respectively. The drug concentrations of tumor were further determined at 12 h post PL-Dox-Ce6 administration (* *p* < 0.05). (**B**) Time course of Ce6 and Dox concentrations in the tumor, liver and heart of C26 tumor-bearing mice without or with (100 J/cm^2^) light irradiation at 2 h after PL-Dox-Ce6 injection (Ce6: 1.75 mg/kg; Dox: 8.31 mg/kg). Data are expressed as the mean ± S.D. for each group (*N* = 3).

It has been shown that PDT-mediated vascular effects can enhance the permeability of tumor vessels, which leads to a significant potentiation in tumor control by combining PDT and Doxil [19]. This might explain the similar therapeutic effect found in Figure 1B. The kinetic profile of plasma showed a significant higher level of PL-Dox-Ce6 at 2 h compared to that of 12 h after drug administration (Figure 1A). We speculated that more PL-Dox-Ce6 could extravasate from the plasma into the tumor tissue due to the PDT-mediated vascular leakage at 2 h after drug administration, which directly contributes to its higher bioavailability of liposomal drug and related therapeutic efficacy. To verify the drug level accumulated in tumor tissue related to the plasma kinetic profile at the time point of light irradiation, we further compared the amounts of Dox and Ce6 in mice tumors that received light irradiation at 2 or 6 h after drug administration. Figure 2A shows the amounts of drug accumulation 12 h after PL-Dox-Ce6 administration without or with light irradiation at different time intervals. The mice that received light irradiation at 2 h presented higher levels of Ce6 and Dox accumulation in tumor tissue compared with those that received it at 6 h. There was no significant difference at the drug amounts in tumors between the mice without light irradiation and those that received light irradiation at 6 h, suggesting that irradiation at 6 h did not have a significant benefit from light irradiation. This phenomenon is more likely corresponding with the plasma levels of Ce6 and Dox, which are significantly higher at 2 h than at 6 h after drug accumulation (Figure 1A). Meanwhile, the biodistribution of PL-Dox-Ce6 in various tissues, including the tumor tissue, heart and liver showed significantly different profiles with and without light irradiation at 2 h after drug administration (Figure 2B and Table S2, Supplementary Materials). As expected, the amounts of Dox and Ce6 in the tumor were clearly higher in the mice that received light irradiation, whereas the mice treated with PL-Dox-Ce6 without light irradiation presented a higher accumulation of both drugs in other organs (heart and liver), suggesting that lower toxicity on normal tissues could be expected in the mice receiving light irradiation at 2 h after drug administration. 

### 3.3. Irradiation Scheme on the Therapeutic Efficiency 

**Figure 3:**
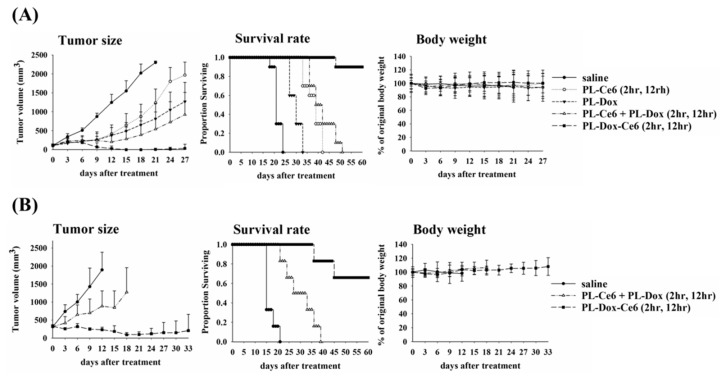
Therapeutic efficacy of C26 tumor-bearing mice that received a single dose of PL-Dox-Ce6 and two doses of light irradiation. One dose of PL-Dox-Ce6 (Ce6: 1.75 mg/kg; Dox: 8.31 mg/kg) was administrated intravenously into tumor-bearing mice. Two doses of light irradiation (100 J/cm^2^) were applied onto the tumor at 2 and 12 h post drug administration. (**A**) The average size of initial tumor was 100 mm^3^ before drug administration (*N* = 10). (**B**) The average size of initial tumor was 300 mm^3^ before drug administration (*N* = 6). Left panel, tumor size; Middle panel, survival rate; Right panel, body weight. The results are represented as the mean ± S.D. for each group.

Previously, we have shown that light irradiation could facilitate the release of liposome-entrapped Dox [20]. As light irradiation at 2 h could significantly increase the amounts of liposomal drugs at tumor, we further explored whether a second light irradiation could be used to improve the therapeutic efficacy of PL-Dox-Ce6. For comparison, PEGylated liposomal Ce6 (PL-Ce6) and liposomal Dox (PL-Dox) were also prepared using the same phospholipid composition. A single dose of PL-Ce6, PL-Dox, a combination of PL-Ce6 and PL-Dox, or PL-Dox-Ce6 in saline was given to the C26 tumor-bearing mice with a tumor size around 100 mm^3^. Two doses of light (100 J/cm^2^) were applied onto the tumor at 2 and 12 h after drug administration, respectively. As shown in the left panel of Figure 3A, PL-Ce6 or PL-Dox alone, PL-Ce6 and PL-Dox in combination as well as the PL-Dox-Ce6 treatments all caused significant suppression of tumor growth compared to the control group with saline administration. Compared to light irradiation at 2 h only (Figure 1B), the therapeutic outcome is significantly higher in mice that received light irradiation at 2 h and 12 h. The most significant improvement was found in the group that received PL-Dox-Ce6, in which tumor-free mice were found after the treatment. Complete tumor regression was observed in 9/10 mice treated with PL-Dox-Ce6, resulting in an apparent cure with clear superiority over the treatment of PL-Dox and PL-Ce6 alone or in combination. The therapeutic results correlated with the survival curves of Kaplan–Meier plot for different treatment groups (middle panel of Figure 3A). There was no significant change of body weight in each study group during the treatment (right panel of Figure 3A). Similar therapeutic effects were also found in a xenograft model on nu/nu nude mice bearing human H460 human lung cancer cells (Figure S1, Supplementary Materials).

### 3.4. Treatment for a Larger Tumor Model 

Large tumors are more difficult to treat than small ones, in part because of the resulting increase in interstitial pressure, which prevents access of drugs to the necrotic core. Thus, we further investigated whether PL-Dox-Ce6 combined with two doses of light irradiation could increase the overall survival of mice with tumor size around 300 mm^3^. As shown in Figure 3B, complete tumor regression was observed in 4/6 mice treated with PL-Dox-Ce6, compared to that of PL-Ce6 and PL-Dox in combination. These results demonstrate that PL-Dox-Ce6 combined with two doses of light irradiation could significantly increase the therapeutic efficacy of PL-Dox-Ce6 while reducing its toxicity.

### 3.5. The Release Profile of Bioavailable Drug after Light Irradiation 

**Figure 4:**
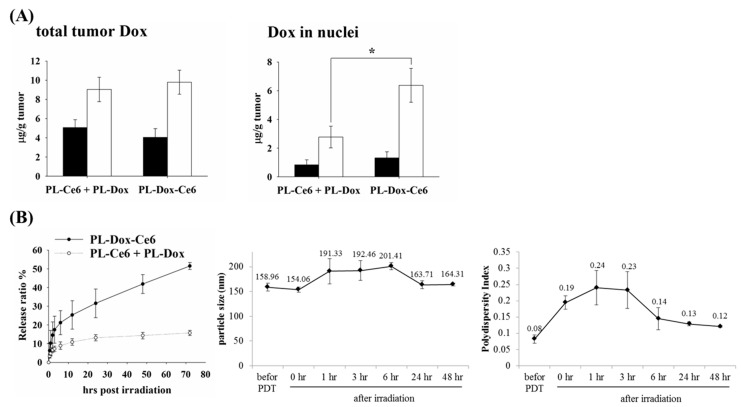
(**A**) Total tumor Dox and Dox in tumor nuclei were determined in tumor-bearing mice after light irradiation. C26-bearing mice were intravenously injected with PL-Dox and PL-Ce6 in combination or PL-Dox-Ce6 (Ce6: 1.75 mg/kg; Dox: 8.22 mg/kg), then received one dose of light irradiation (100 J/cm^2^) 2 h after drug administration (*N* = 3). The levels of total tumor Dox (left panel) and Dox in the tumor nuclei (right panel) were analyzed 4 h after drug administration. ■: tumor without light irradiation; □: tumor with light irradiation (* *p* < 0.05). (**B**) The release profile, alterations of particle size and polydispersities (PDI) of PL-Dox-Ce6 after light irradiation (10 J/cm^2^). Left panel, the kinetics of Dox released from PL-Dox-Ce6 (●) or PL-Dox and PL-Ce6 in combination (○) after light irradiation. The mean size (Middle panel) and related PDI (Right panel) of PL-Dox-Ce6 at different time points after light irradiation. The results are represented as the mean ± S.D. for each group.

As shown above, PL-Dox-Ce6 exert significant therapeutic efficacy than the combination of PL-Dox and PL-Ce6. We further examined whether the increased therapeutic efficacy relates to the higher amounts of liposomal drugs in the tumor. As shown in the left panel of Figure 4A, a higher amount of Dox could be found in the tumor tissue that received light irradiation. However, there was no difference in the increased amount of Dox deposited in the tumor tissue after light irradiation between the groups treated with PL-Dox-Ce6 and the combination of PL-Dox and PL-Ce6. This result indicates that light irradiation at 2 h in both groups could photodamage the vascular endothelial linings of tumor tissue, resulting in an enhanced permeability and retention of liposomal drugs in the tumor. It has been known that the cytotoxic action of Dox is in the nucleus to intercalate into DNA, forming DNA adducts and inhibiting topoisomerase II. In this regard, we further measured the levels of Dox in the tumor nuclei that allow therapeutic activity to be correlated with the bioavailable drug levels. As shown in the right panel of Figure 4A, we found that the nucleus amounts of Dox in the PL-Dox-Ce6 treated group maintained a two-fold enhancement compared to that of PL-Dox and PL-Ce6 in combination. The significant increase of Dox in the nuclei of tumor cells suggests that light irradiation not only photodamaged the tumor vascular but also significantly enhanced the Dox release from the liposomal PL-Dox-Ce6. On the contrary, the increased level of Dox in the nuclei of tumor cells was lower in animals treated with PL-Dox and PL-Ce6 in combination, suggesting a higher bioavailability of Dox in PL-Dox-Ce6. This argument was further supported by an in vitro kinetic study. 

Previously, we have shown that the drug leakage of PL-Dox-Ce6 was less than 3% in the first three days without light irradiation [20].). However, the release of encapsulated Dox from the PL-Dox-Ce6 is significantly higher, from 10% to 40% after light irradiation, compared to that of PL-Ce6 and PL-Dox in combination (left panel of Figure 4B). The significant increase in Dox release indicated that PL-Dox-Ce6 became leaky, resulting in Dox release and further concentrate into the nucleus after light irradiation. Interestingly, a significant but transient change was found in the particle size and related PI value of PL-Dox-Ce6 after light irradiation (middle and right panel of Figure 4B). This alteration suggests the possible reconstruction of liposomal particles under photodynamic effect. Combined together, the results for the bioavailable drug levels in tumor tissue well explained the significant therapeutic effects in using PL-Dox-Ce6.

### 3.6. Characteristics and Stability of PL-cDDP-Ce6 Liposomes

**Figure 5:**
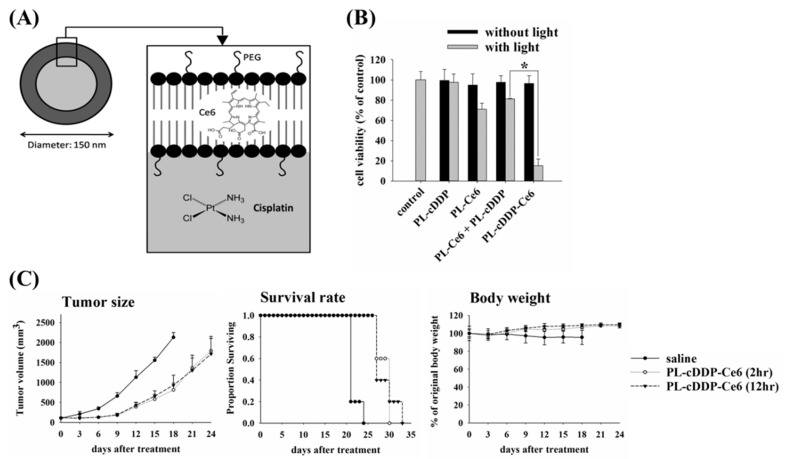
(**A**) Diagram of dual-effect liposome (PL-cDDP-Ce6) with cisplatin (cDDP) and Ce6 encapsulated in the interior and lipid bilayer, respectively. (**B**) In vitro cytotoxicity under the impact of different liposomal drugs. A375 cells were incubated with PL-Dox-Ce6, PL-cDDP, PL-Ce6, and the combination of PL-Ce6 and PL-cDDP. The concentration of Ce6 and cDDP is 0.5 and 1.12 mg/mL, respectively. After incubation for 2 h, cells were irradiated with light (0.1 J/cm^2^) and cell viability was determined by MTT assay 24 h after light irradiation (* *p* < 0.05). (**C**) Tumor growth control of PL-cDDP-Ce6 (Ce6: 1.75 mg/kg; cisplatin: 3.72 mg/kg). After drug injection, light irradiation (100 J/cm^2^) was given at 2 h and 12 h, respectively. Left panel, tumor size; Middle panel, survival rate; Right panel, body weight. The results are represented as the mean ± S.D. for each group (*N* = 5).

In the above studies, the PL-Dox-Ce6 combined with two doses of light irradiation has shown its excellent therapeutic efficacy. We then further examined whether this dual-effect liposome and its therapeutic scheme could be applied to other chemotherapeutic drugs. To verify this, cisplatin (cDDP) and Ce6 were co-encapsulated in the PEGylated liposome (named as PL-cDDP-Ce6), in which Ce6 was located in the lipid bilayer and cDDP in the aqueous interior. The schematic diagram of the PL-cDDP-Ce6 is shown in Figure 5A. For comparison, PL-Ce6 and PEGylated liposomal cDDP (PL-cDDP) were also prepared using the same phospholipid composition. The characteristics of liposomal drugs are listed in Table S3, Supplementary Materials. The particle size of both PL-Ce6 and PL-cDDP-Ce6 was around 150 nm, and it was 132 nm for PL-cDDP. Their lipid recovery was all above 80%. The entrapment efficiency of Ce6 in PL-Ce6 and PL-cDDP-Ce6 was similar (more than 80%). Interestingly, the entrapment efficiency of cDDP in this dual-effect liposome was 5% higher than in PL-cDDP. The stability of PL-cDDP-Ce6 at 4 °C is listed in Table S4, Supplementary Materials. There was no significant difference in particle size within two months. The leakage of cDDP and Ce6 was about 5% within 3 days. However, about 10% leakage was found when stored for 2 months. As found in PL-Dox-Ce6 (Figure 4B), PL-cDDP-Ce6 showed a similar in vitro release profile after light irradiation (Figure S2A, Supplementary Materials). The release of encapsulated cDDP from the PL-cDDP-Ce6 is significantly higher from 10%–25% after light irradiation compared to that of PL-Ce6 and PL-cDDP in combination. Meanwhile, a similar reconstitution of liposomal particle was also found in PL-cDDP-Ce6 (Figure S2B, Supplementary Materials). 

### 3.7. The Cytotoxicity of PL-cDDP-Ce6 In Vitro and In Vivo

The cytotoxicity of PL-cDDP-Ce6 was first determined in vitro via MTT assay. No toxicity was observed when these cells were treated with all of these liposomal drugs in the dark (Figure 5B). After light irradiation, there was about 80% cellular viability in the group of cells treated with PL-Ce6 and the mixture of PL-Ce6 and PL-cDDP. In contrast, the cellular viability was less than 20% under the treatment of PL-cDDP-Ce6 after light irradiation (Figure 5B). These results indicate that PL-cDDP-Ce6 could exert significant cytotoxicity compared to the combined use of PL-Ce6 and PL-cDDP after light irradiation.

**Figure 6:**
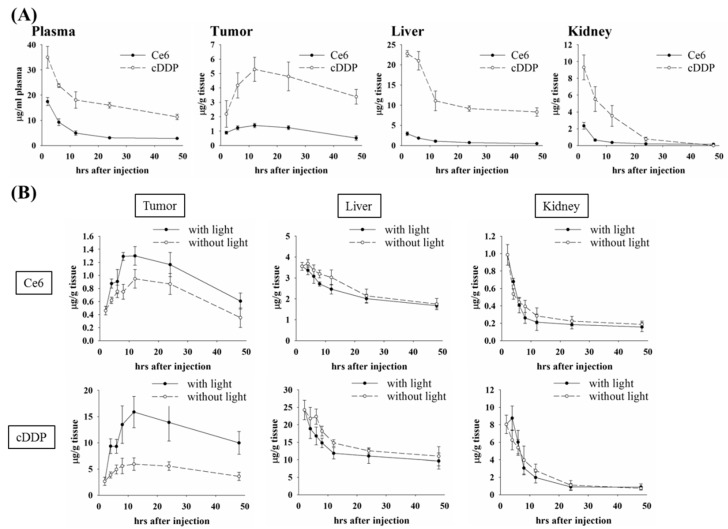
Biodistribution of drugs in C26 tumor-bearing mice after intravenous administration of PL-cDDP-Ce6 (Ce6: 1.75 mg/kg and cDDP: 3.54 mg/kg). (**A**) The concentrations of Ce6 and cDDP in plasma, tumor, liver and kidney at different time points after injection of PL-cDDP-Ce6. Results are expressed as the mean ± S.D. for *N* = 3 at each time point. (**B**) The concentrations of Ce6 and cDDP in the tumor, liver and kidney of C26 tumor-bearing mice with or without light irradiation at 2 h after drug administration. Results are expressed as the mean ± S.D. for *N* = 3 at each time point.

**Figure 7:**
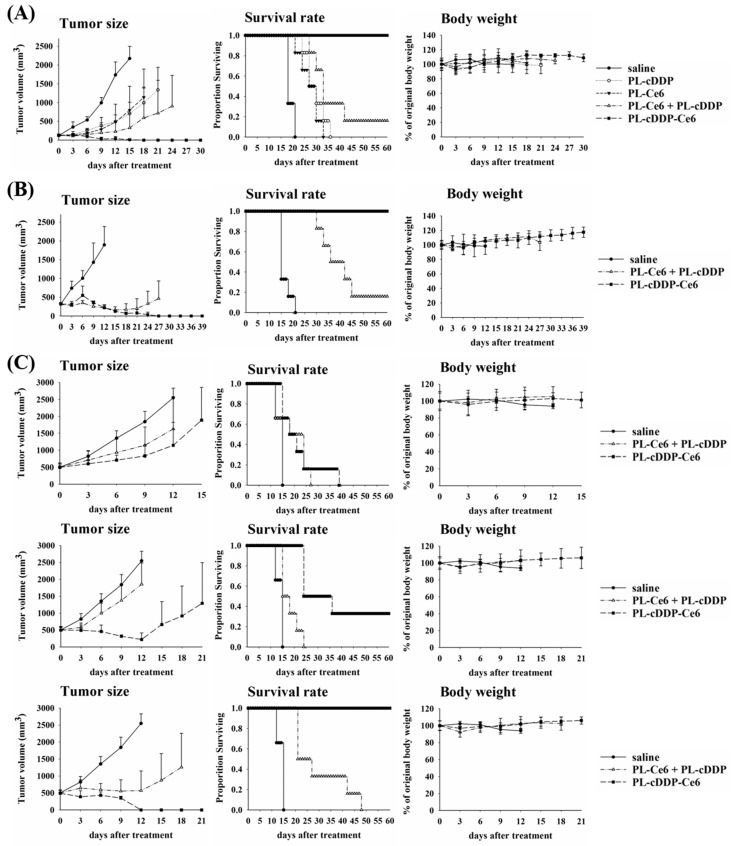
Therapeutic efficacy of mice bearing different sizes of C26 tumor. A single dose of PL-cDDP-Ce6 was intravenously injected to the animals of each group (*N* = 6). After drug administration, two doses of light irradiation (100 J/cm^2^) were applied onto the tumor at 2 and 12 h. (**A**) The average size of the initial tumor was 100 mm^3^ before drug administration (Ce6: 1.75 mg/kg; cDDP: 3.73 mg/kg). (**B**) The average size of the initial tumor was 300 mm^3^ before drug administration (Ce6: 1.75 mg/kg; cDDP: 3.87 mg/kg). Left panel, tumor size; Middle panel, Survival rate; Right panel, body weight. (**C**) Survival rate of PL-cDDP-Ce6 increases in a dose-dependent manner in mice bearing a tumor with an average initial size of 500 mm^3^. The dose of Ce6 administrated in mice was 1.75 mg/kg and the dose of cDDP varied under the doses of 3.83 mg/kg (upper panel), 4.75 mg/kg (middle panel) and 5.71 mg/kg (lower panel). The results are represented as the mean ± S.D. for each group.

We then further examined the in vivo therapeutic efficacy of PL-cDDP-Ce6 in BALB/c mice bearing a C26 tumor. A single dose of PL-cDDP-Ce6 was intravenously administered into the mice bearing a C26 tumor of 100 mm^3^ and received light irradiation either at 2 h or 12 h after drug injection, respectively. Compared to the control group, a significant reduction of the mean tumor sizes and increased survival rate were found in PL-cDDP-Ce6 treated groups (Figure 5C). However, we did not find significant difference in tumor suppression and survival rate between the groups that received light irradiation either at 2 h or 12 h, which is the same as the therapeutic study using PL-Dox-Ce6 (Figure 2B). The kinetic profiles of PL-cDDP-Ce6 were similar to those of PL-Dox-Ce6 (Figure 6A and Table S5, Supplementary Materials). We again examined the liposomal drug content in different tissues with or without light irradiation at 2 h post drug administration. As expected, the amounts of cDDP and Ce6 in tumor were significantly higher in mice that received light irradiation (Figure 6B and Table S6, Supplementary Materials). Meanwhile, mice treated with liposomal drugs without light irradiation presented a higher accumulation of Ce6 and cDDP in other organs such as liver and kidney, suggesting that lower toxicity on normal tissues could be expected in the mice receiving the light irradiation at 2 h post drug administration. Furthermore, the therapeutic efficacy of PL-cDDP-Ce6 was examined in the C26 syngeneic BALB/c mice. A single dose of PL-Ce6, PL-cDDP, combination of PL-Ce6 and PL-cDDP, or PL-cDDP-Ce6 in saline was given to the mice with different tumor sizes, then two doses of light (100 J/cm^2^) were applied onto the tumor at 2 and 12 h post drug administration, respectively. As shown in Figure 7A,B, mice with tumor sizes of either 100 or 300 mm^3^ became tumor-free in the group treated with one dose of PL-cDDP-Ce6 (1.75 mg/kg of Ce6 and 3.7~3.8 mg/kg of cDDP), with clear superiority over the treatment of PL-Ce6 and PL-cDDP alone or in combination. Then, the therapeutic efficacy of this PL-cDDP-Ce6 was further examined in mice with a tumor size around 500 mm^3^. The overall survival could significantly increase when the drug dose of cDDP in the PL-cDDP-Ce6 increased from 3.83 mg/kg to 5.71 mg/kg (Figure 7C). In fact, complete tumor regression was observed in the group treated with PL-cDDP-Ce6 (1.75 mg/kg of Ce6 and 5.71 mg/kg of cDDP), compared to that of PL-Ce6 and PL-cDDP in combination. There was no significant change of body weight in each study groups during the treatment. Similar results were also found in nude mice bearing human H460 xenograft with a tumor size of 500 mm^3^ (Figure S3, Supplementary Materials). These results demonstrate that these dual-effect liposomes combined with two doses of light irradiation could significantly increase the therapeutic efficacy while reducing its toxicity. 

## 4. Discussion

In this study, we first aimed to perform a thorough in vivo pharmacokinetics and biodistribution study of a dual-effect liposomes co-encapsulated photosensitizer and chemotherapeutic drugs. The therapeutic efficacy was further determined at different drug-light intervals based on the kinetic data of plasma and tumor tissue. The most significant finding is that one dose of these liposomal drugs combined with two doses of light irradiation significantly increased the therapeutic efficacy and the life span of tumor bearing mice. In the future, it will be necessary to further address whether there is systemic toxicity in using this dual-effect liposome, which is important for its clinical development. For the light irradiation, it should be noted that it is not simply a matter of performing a second irradiation at some arbitrary time point after the first irradiation. We performed light irradiation 2 and 12 h post drug irradiation based on the following two reasons. First, the pharmacokinetic profile (Figure 1A) showed that the maximum concentration of drugs in plasma is at 2 h after liposomes injection. Light irradiation at this time point can significantly increase the liposomal drug content in tumors due to PDT-induced vascular leakage, as shown in Figure 2A. Secondly, the PK data showed that liposomal drug levels in tumors reached a peak at 12 h at which a better therapeutic efficacy was expected with light irradiation. The time interval between the light fractions should be based on the PK profile of liposomal drug to confer a clinical advantage of combining PDT and chemotherapy. Hence, this study establishes a platform that utilizes the dual-effect liposome combined with an optimal illumination scheme to improve the therapeutic outcomes.

The outcome of vascular damage induced by PDT depends on the photosensitizer doses. It has been demonstrated that PDT mediated by a high dose of photosensitizer resulted in permanent vessel occlusion, causing effective suppression of tumor growth [17,23,24]. Low-dose photosensitizer used in PDT regimen can enhance vascular permeability without causing acute vascular shutdown, which has been further developed to deliver macromolecular drug into tumors [19,25,26,27]. In this study, the increasing amounts of Dox in tumor tissue could be found in mice treated with PL-Dox-Ce6 or the combined use of PL-Dox and PL-Ce6 (Figure 4A), indicating vascular leakage induced by PDT. However, the present work differs from the published studies by the use of the dual-effect liposome in which Ce6 and chemotherapeutic drug (doxorubicin or cisplatin) are co-encapsulated in the same carrier but not the combination of two liposomal drugs. Using these dual-effect liposomes, two different drugs could be delivered to the target tissue simultaneously. Furthermore, light-irradiation could significantly increase the bioavailable Dox in the cell nuclei of tumor tissue in animals treated with dual-effect liposome (Figure 4A). This could be further supported from the in vitro release study, which shows a four to five folds increase of chemotherapeutic drug released from the dual-effect liposome compared to the combined use of two liposomal drugs after light irradiation (Figure 4B and Figure S2A, Supplementary Materials). The significant increase of bioavailable drug released from liposome and deposited in the tumor is likely an important factor for the therapeutic efficacy of using dual-effect liposome.

PEGylated liposome has been used to reduce non-specific cytotoxicity and RES uptake in chemotherapy. However, the delayed release of drug from the stealth liposome limits its therapeutic efficacy in clinical practice [9]. In this study, we showed that light irradiation could destabilize the dual-effect liposome to enhance the sustained release of chemotherapeutic drug into the nucleus to exert its therapeutic efficacy. Meanwhile, a significant reduction of chemotherapeutic drug presebt in normal tissues was also found after the first light irradiation (Figure 2B and Figure 6B). Therefore, a lower side-effect on normal tissue could be expected in mice receiving the dual-effect liposome during the treatment. These results further justify the advantage of using dual-effect liposome in combining PDT and chemotherapy for tumor treatment.

Combination therapy by combing two or multiple therapeutic agents has been developed for treating cancer with synergistic efficacy while lowering the doses of each drug to reduce side effects. However, the various pharmacokinetic profiles of different drugs might lead to inconsistent pharmacokinetics and biodistribution and therefore, an unfavorable treatment outcome. Recently, a nanocarrier-based delivery system has been developed to co-encapsulate multiple therapeutic agents with unified pharmacokinetics and enhancing therapeutic efficacy [28,29]. Except for the increased therapeutic efficacy and reduced side-effect, this dual-effect liposome has other pivotal advantages used for combinational therapy, such as prolonged circulation, controlled-release profile in tumor tissue, and normalized pharmacokinetics and pharmacodynamics of the therapeutic agents.

## 5. Conclusions

In summary, here, we demonstrated that a single-dose of dual-effect liposome combined with an optimal illumination scheme could increase the tumor disposition of bioavailable drugs formulated in liposome while reducing exposure to healthy organs. The vascular damage and significant increase in the bioavailable concentration of chemotherapeutic drugs are the two main factors attributed to the complete tumor eradication, which could only be found in the tumor-bearing mice treated with dual-effect liposomes but not those treated with two liposomal drugs in combination.

## Supplementary Materials

The following are available online at https://www.mdpi.com/1999-4923/11/11/617/s1, Figure S1: Therapeutical efficacy of PL-Dox-Ce6 on nu/nu nude mice bearing human H460 human lung cancer cells, Figure S2: Release profile, alterations of particle size and polydispersities (PDI) of PL-cDDP-Ce6 after light irradiation, Figure S3: Therapeutical efficacy of PL-cDDP-Ce6 on nu/nu nude mice bearing H460 tumors with average tumor size of 500 mm^3^ before drug administration, Table S1: Biodistribution of Dox and Ce6 at different time points in BALB/c mice bearing syngeneic C26 tumor after intravenous injection of PL-Dox-Ce6, Table S2: Time course of Ce6 and Dox concentrations in different tissues of C26 tumor-bearing mice without or with light irradiation at 2 hr after intravenous injection of PL-Dox-Ce6, Table S3: Particle size and entrapment of PL-Ce6, PL-cDDP and PL-cDDP-Ce6, Table S4: Stability and particle size of PL-cDDP-Ce6 at 4 °C, Table S5: Concentration of cDDP and Ce6 at different time points in BALB/c mice bearing syngeneic C26 tumor after intravenous injection of PL-cDDP-Ce6, Table S6: Time course of Ce6 and cDDP concentrations in different tissues of C26 tumor-bearing mice without or with light irradiation at 2 hr after intravenous injection of PL-cDDP-Ce6.
