# Supplementary material for: Co-Encapsulation of Chlorin e6 and Chemotherapeutic Drugs in a PEGylated Liposome Enhance the Efficacy of Tumor Treatment: Pharmacokinetics and Therapeutic Efficacy
Source: Pharmaceutics. 2019 Nov 17;11(11):617. doi: 10.3390/pharmaceutics11110617 (PMC6920861; doi:10.3390/pharmaceutics11110617)
Supplement: Supplementary file 1 [file pharmaceutics-11-00617-s001.pdf]

pharmaceutics

MDPI

Supplementary Materials: Co-Encapsulation of Chlorin e6 and Chemotherapeutic Drugs in a PEGylated Liposome Enhance the Efficacy of Tumor Treatment: Pharmacokinetics and Therapeutic Efficacy

Po-Chun Peng [1], Ruey-Long Hong [2], Tsuimin Tsai [3] and Chin-Tin Chen [1,*]

Table S1. Biodistribution of Dox and Ce6 at different time points in BALB/c mice bearing syngeneic C26 tumor after intravenous injection of PL-Dox-Ce6 (*N* = 3).

Hours after injection	Plasma (g/mL)	Tumor (g/g)	Liver (g/g)	Heart (g/g)	Kidney (g/g)	Lung (g/g)	Muscle (g/g)	Skin (g/g)
**Ce6**								
2 h	12.2 ± 11.45	0.47 ± 0.08	3.63 ± 0.16	0.24 ± 0.08	1.20 ± 0.37	0.42 ± 0.10	0.03 ± 0.01	0.19 ± 0.02
6 h	4.47 ± 0.32	0.87 ± 0.14	3.17 ± 0.08	N/A	0.72 ± 0.13	0.08 ± 0.02	N/A	0.05 ± 0.01
12 h	2.05 ± 0.20	0.97 ± 0.10	2.81 ± 0.27	N/A	0.48 ± 0.05	0.06 ± 0.03	N/A	0.04 ± 0.02
24 h	1.36 ± 0.21	0.91 ± 0.12	1.66 ± 0.09	N/A	0.16 ± 0.07	0.01 ± 0.00	N/A	N/A
48 h	0.19 ± 0.02	0.24 ± 0.05	1.25 ± 0.09	N/A	N/A	N/A	N/A	N/A
**Dox**								
2 h	51.57 ± 3.57	2.41 ± 0.60	21.53 ± 3.48	0.71 ± 0.11	5.10 ± 1.11	3.11 ± 0.36	0.62 ± 0.21	1.14 ± 0.26
6 h	29.43 ± 2.61	6.26 ± 1.60	28.52 ± 2.97	0.14 ± 0.07	4.83 ± 0.70	2.27 ± 0.22	0.39 ± 0.24	0.91 ± 0.07
12 h	17.09 ± 1.25	8.81 ± 1.10	33.64 ± 1.56	0.10 ± 0.08	4.39 ± 0.28	2.16 ± 0.10	0.30 ± 0.12	0.80 ± 0.12
24 h	8.94 ± 2.74	8.74 ± 1.68	19.62 ± 0.92	0.01 ± 0.04	2.73 ± 0.90	0.67 ± 0.21	0.02 ± 0.10	0.79 ± 0.06
48 h	1.67 ± 0.91	2.60 ± 0.70	18.70 ± 1.46	-0.04 ± 0.08	0.75 ± 0.09	0.35 ± 0.15	0.02 ± 0.07	0.58 ± 0.12

Table S2. Time course of Ce6 and Dox concentrations in different tissues of C26 tumor-bearing mice without or with light irradiation at 2 h after intravenous injection of PL-Dox-Ce6 (*N* = 3).

Group	Drug	Hours after injection	Plasma (g/mL)	Tumor (g/g)	Liver (g/g)	Heart (g/g)	Spleen (g/g)	Kidney (g/g)	Lung (g/g)
without light	Ce6	2 h	6.65 ± 0.79	0.28 ± 0.08	1.62 ± 0.26	0.14 ± 0.01	3.63 ± 0.08	0.96 ± 0.07	0.27 ± 0.07
		4 h	5.35 ± 0.22	0.43 ± 0.07	2.11 ± 0.06	0.06 ± 0.01	4.17 ± 0.23	0.82 ± 0.04	0.18 ± 0.03

		6 h	4.00 ± 0.62	0.53 ± 0.03	2.25 ± 0.39	0.04 ± 0.00	4.15 ± 0.08	0.58 ± 0.13	0.12 ± 0.02
		8 h	3.55 ± 0.45	0.59 ± 0.09	2.27 ± 0.20	0.02 ± 0.01	4.35 ± 0.14	0.38 ± 0.06	0.07 ± 0.03
		12 h	2.53 ± 0.98	0.67 ± 0.04	2.16 ± 0.35	0.02 ± 0.02	3.95 ± 0.40	0.31 ± 0.07	0.04 ± 0.02
		24 h	0.87 ± 0.07	0.57 ± 0.08	2.09 ± 0.09	N/A	3.32 ± 0.29	0.11 ± 0.04	0.04 ± 0.01
		48 h	0.72 ± 0.07	0.30 ± 0.08	1.04 ± 0.20	N/A	2.07 ± 0.10	0.06 ± 0.02	N/A
	**Dox**	2 h	60.16 ± 6.64	2.45 ± 0.30	15.08 ± 1.54	1.52 ± 0.04	27.20 ± 0.60	5.38 ± 0.63	3.96 ± 0.69
		4 h	51.54 ± 3.31	5.36 ± 1.42	24.52 ± 0.40	1.39 ± 0.04	29.77 ± 0.91	5.01 ± 0.92	3.55 ± 0.55
		6 h	46.85 ± 2.15	7.33 ± 1.05	27.56 ± 4.31	1.15 ± 0.01	33.42 ± 0.33	5.44 ± 0.76	3.32 ± 0.30
		8 h	40.47 ± 5.86	8.48 ± 1.05	28.76 ± 2.00	0.91 ± 0.12	33.55 ± 1.44	4.72 ± 1.12	3.27 ± 0.30
		12 h	31.63 ± 4.92	9.57 ± 1.70	29.92 ± 3.67	0.79 ± 0.18	35.96 ± 0.92	4.31 ± 0.79	2.78 ± 0.67
		24 h	18.82 ± 5.69	8.96 ± 1.68	32.84 ± 0.97	0.59 ± 0.16	29.41 ± 1.77	3.80 ± 0.29	1.82 ± 0.16
		48 h	13.45 ± 1.05	3.98 ± 1.09	24.87 ± 1.55	0.38 ± 0.12	24.29 ± 1.49	2.21 ± 0.27	0.58 ± 0.36
**with light**	**Ce6**	4 h	3.97 ± 0.46	0.79 ± 0.11	2.21 ± 0.11	0.06 ± 0.01	4.08 ± 0.44	0.52 ± 0.05	0.16 ± 0.02
		6 h	3.59 ± 0.41	0.75 ± 0.16	2.02 ± 0.23	0.01 ± 0.01	3.77 ± 0.18	0.44 ± 0.04	0.09 ± 0.03
		8 h	3.12 ± 0.17	0.83 ± 0.11	1.93 ± 0.13	0.01 ± 0.01	3.68 ± 0.31	0.35 ± 0.07	0.07 ± 0.03
		12 h	2.18 ± 0.21	1.06 ± 0.15	1.72 ± 0.26	0.01 ± 0.01	3.19 ± 0.01	0.19 ± 0.04	0.06 ± 0.04
		24 h	0.67 ± 0.01	1.09 ± 0.12	1.59 ± 0.15	N/A	2.65 ± 0.22	0.04 ± 0.01	0.02 ± 0.01
		48 h	0.19 ± 0.06	0.88 ± 0.05	0.88 ± 0.19	N/A	1.91 ± 0.11	N/A	0.01 ± 0.02
	**Dox**	4 h	48.69 ± 3.36	10.98 ± 1.02	27.67 ± 2.06	1.26 ± 0.03	33.13 ± 2.33	5.21 ± 0.23	3.42 ± 0.45
		6 h	45.52 ± 1.49	10.55 ± 1.97	28.51 ± 2.56	1.02 ± 0.14	31.20 ± 2.67	5.11 ± 0.68	3.41 ± 0.46
		8 h	39.40 ± 5.68	12.74 ± 1.12	27.15 ± 1.44	0.58 ± 0.16	30.89 ± 1.94	4.68 ± 1.05	3.02 ± 0.57
		12 h	28.58 ± 3.07	15.29 ± 2.10	28.48 ± 3.18	0.34 ± 0.10	29.89 ± 2.70	4.58 ± 0.49	2.69 ± 0.35
		24 h	14.93 ± 0.60	17.93 ± 1.10	29.02 ± 1.85	0.37 ± 0.11	25.58 ± 2.34	3.23 ± 0.38	1.99 ± 0.41
		48 h	7.49 ± 0.80	18.30 ± 0.91	20.36 ± 1.06	0.23 ± 0.10	22.70 ± 1.38	2.30 ± 0.19	0.48 ± 0.21

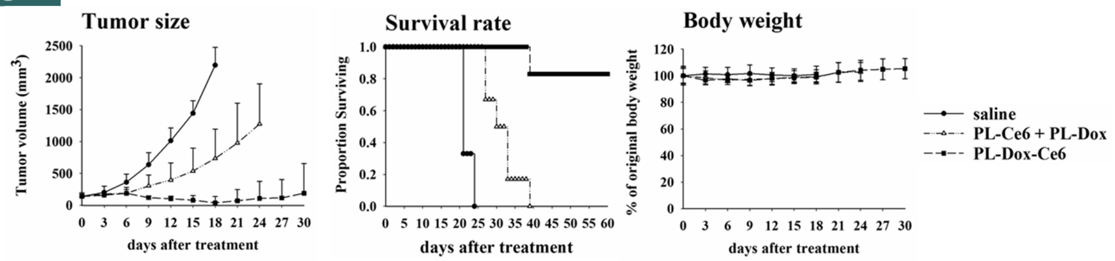

Figure S1. Therapeutical efficacy of PL-Dox-Ce6 on nu/nu nude mice bearing human H460 human lung cancer cells. After liposomal drug (Ce6: 1.75 mg/kg and Dox: 8.2 mg/kg) injection, light irradiation was applied onto the tumor at 2 h and 12 h, respectively. Left panel, tumor size; Middle panel, survival rate; Right panel, body weight. The results are represented as the mean ± S.D. for each group ($N = 6$). The mean tumor size in PL-Dox-Ce6 treating groups were significantly smaller than the control-saline group ($P < 0.001$). Complete tumor regression was observed in 5/6 mice treated with PL-Dox-Ce6, resulting in apparent cure with clear superiority over the treatment of PL-Dox and PL-Ce6 in combination.

Table S3. Particle size and entrapment of PL-Ce6, PL-cDDP and PL-cDDP-Ce6 ($N = 3$).

**Liposome**	**Particle size (nm)**	**Lipid recovery (%)**	**Ce6 entrapment (g/mol)**	**Ce6 entrapment efficiency (%)**	**cDDP entrapment (g/mol)**	**cDDP entrapment efficiency (%)**
**PL-Ce6**	150 ± 29.7	83.5 ± 2.5	6.8 ± 0.2	87 ± 2.8		
**PL-cDDP**	132.6 ± 27.3	82 ± 3.1			11.9 ± 1.3	15.1 ± 1.7
**PL-cDDP-Ce6**	147.3 ± 31.5	81.5 ± 2.1	6.6 ± 0.2	84.8 ± 2	13.9 ± 1.2	17.7 ± 1.5

Table S4. Stability and particle size of PL-cDDP-Ce6 at 4°C ($N = 3$).

**Stability of PL-cDDP-Ce6**	**Days**							
	**0**	**1**	**3**	**7**	**14**	**21**	30	60
Leakage of Ce6 (%)	0.00	2.7 ± 1.1	5 ± 1.9	5.5 ± 2.1	4.9 ± 2.2	9.1 ± 2.7	11.1 ± 1.7	12.5 ± 1.5
Leakage of cDDP (%)	0.00	4.8 ± 0.6	5.7 ± 1	5.8 ± 1.8	8.3 ± 1.8	6.8 ± 1.7	10.2 ± 0.9	12.4 ± 0.9
Particle size (nm)	148 ± 10			145 ± 5	148 ± 9	150 ± 7	150 ± 9	150 ± 14

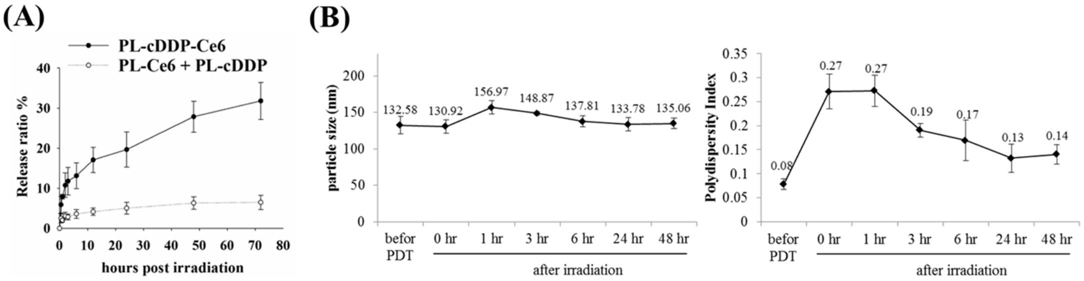

Figure S2. Release profile, alterations of particle size and polydispersities (PDI) of PL-cDDP-Ce6 after light irradiation (10 J/cm²). (**A**) the kinetics of cDDP released from PL-cDDP-Ce6 (●) or PL-cDDP and PL-Ce6 in combination (○) after light irradiation. (**B**) Mean size (Middle panel) and related PDI (Right

panel) of PL-cDDP-Ce6 at different time points after light irradiation. The results are represented as the mean ± S.D. for each group.

Table S5. Concentration of cDDP and Ce6 at different time points in BALB/c mice bearing syngeneic C26 tumor after intravenous injection of PL-cDDP-Ce6 ($N = 3$).

Hours after injection	Plasma (g/mL)	Tumor (g/g)	Liver (g/g)	Heart (g/g)	Spleen (g/g)	Kidney (g/g)	Lung (g/g)
**Ce6**							
2 h	17.44 ± 1.59	0.90 ± 0.10	2.93 ± 0.44	0.22 ± 0.02	3.63 ± 0.52	2.34 ± 0.40	0.60 ± 0.04
6 h	9.24 ± 1.31	1.22 ± 0.13	1.83 ± 0.10	0.09 ± 0.01	3.25 ± 0.31	0.65 ± 0.12	0.44 ± 0.09
12 h	4.92 ± 0.90	1.39 ± 0.15	1.07 ± 0.22	0.04 ± 0.02	2.92 ± 0.27	0.36 ± 0.02	0.26 ± 0.04
24 h	3.06 ± 0.18	1.24 ± 0.14	0.73 ± 0.07	N/A	2.25 ± 0.48	0.20 ± 0.09	0.19 ± 0.06
48 h	2.82 ± 0.09	0.52 ± 0.16	0.48 ± 0.08	N/A	1.96 ± 0.13	0.15 ± 0.02	0.09 ± 0.07
**cDDP**							
2 h	35.00 ± 4.33	2.19 ± 0.92	22.79 ± 0.78	N/A	12.94 ± 2.24	9.30 ± 1.48	1.52 ± 0.24
6 h	23.80 ± 0.71	4.17 ± 0.87	21.01 ± 2.29	N/A	19.45 ± 3.19	5.52 ± 1.48	1.71 ± 0.52
12 h	18.08 ± 3.24	5.29 ± 0.84	11.08 ± 2.45	N/A	25.99 ± 3.80	3.53 ± 1.24	2.40 ± 0.34
24 h	15.98 ± 1.09	4.80 ± 0.99	9.16 ± 0.66	N/A	14.16 ± 1.12	0.77 ± 0.23	1.57 ± 0.37
48 h	11.35 ± 1.05	3.39 ± 0.51	8.32 ± 1.05	N/A	8.53 ± 0.96	N/A	N/A

Table S6. Time course of Ce6 and cDDP concentrations in different tissues of C26 tumor-bearing mice without or with light irradiation at 2 h after intravenous injection of PL-cDDP-Ce6 ($N = 3$).

Group	Drug	Hours after injection	Plasma (g/mL)	Tumor (g/g)	Liver (g/g)	Kidney (g/g)	Spleen (g/g)
**without light**	**Ce6**	2 h	13.36 ± 1.14	0.46 ± 0.06	3.56 ± 0.19	0.99 ± 0.12	3.94 ± 0.18
		4 h	10.18 ± 0.95	0.62 ± 0.05	3.69 ± 0.19	0.54 ± 0.06	4.19 ± 0.27
		6 h	7.31 ± 1.03	0.75 ± 0.10	3.37 ± 0.25	0.44 ± 0.05	3.86 ± 0.23
		8 h	6.09 ± 1.15	0.75 ± 0.11	3.19 ± 0.19	0.39 ± 0.07	3.79 ± 0.25
		12 h	4.73 ± 0.53	0.95 ± 0.14	3.03 ± 0.36	0.28 ± 0.09	3.56 ± 0.29
		24 h	3.80 ± 0.18	0.87 ± 0.16	2.15 ± 0.33	0.22 ± 0.05	3.13 ± 0.35
		48 h	2.35 ± 0.48	0.35 ± 0.15	1.75 ± 0.27	0.19 ± 0.04	2.62 ± 0.27

	cDDP	2 h	31.02 ± 1.73	2.66 ± 0.79	24.24 ± 2.78	8.05 ± 1.06	15.53 ± 1.93
		4 h	23.80 ± 0.83	3.87 ± 0.60	21.79 ± 3.19	6.25 ± 1.14	17.35 ± 2.50
		6 h	21.10 ± 1.79	4.94 ± 0.86	22.34 ± 2.19	5.38 0.90	17.68 ± 2.15
		8 h	17.47 ± 2.11	5.58 ± 1.45	18.18 ± 1.37	3.97 ± 1.60	20.37 ± 2.47
		12 h	14.17 ± 0.97	5.95 ± 1.17	14.77 ± 0.96	2.77 ± 0.73	21.37 ± 3.60
		24 h	9.84 ± 1.46	5.56 ± 0.82	12.53 ± 0.83	1.10 ± 0.53	17.52 ± 2.47
		48 h	5.83 ± 1.60	3.60 ± 0.76	11.00 ± 2.74	0.74 ± 0.10	14.78 ± 1.52
with light	Ce6	4 h	8.77 ± 0.59	0.87 ± 0.07	3.37 ± 0.21	0.68 ± 0.04	3.54 ± 0.24
		6 h	6.54 ± 0.78	0.91 ± 0.18	3.08 ± 0.33	0.41 ± 0.09	3.35 ± 0.31
		8 h	5.25 ± 0.76	1.29 ± 0.06	2.72 ± 0.10	0.26 ± 0.06	3.18 ± 0.19
		12 h	4.90 ± 0.54	1.30 ± 0.14	2.46 ± 0.23	0.21 ± 0.09	3.16 ± 0.23
		24 h	3.80 ± 0.64	1.16 ± 0.18	2.01 ± 0.21	0.19 ± 0.05	2.81 ± 0.20
		48 h	1.82 ± 0.33	0.61 ± 0.12	1.67 ± 0.10	0.16 ± 0.05	2.53 ± 0.14
	cDDP	4 h	20.09 ± 1.67	9.34 ± 1.39	18.87 ± 2.79	8.76 ± 1.39	17.97 ± 1.89
		6 h	19.08 ± 1.16	9.29 ± 1.33	16.80 ± 2.58	6.01 ± 1.33	13.44 ± 0.80
		8 h	16.64 ± 3.21	13.45 ± 3.57	14.81 ± 1.37	3.06 ± 0.77	14.52 ± 1.87
		12 h	12.04 ± 2.55	15.87 ± 2.99	11.81 ± 1.64	1.97 ± 0.61	14.61 ± 2.38
		24 h	8.27 ± 1.94	13.88 ± 3.57	11.03 ± 2.07	0.89 ± 0.39	13.93 ± 1.90
		48 h	4.84 ± 0.68	9.96 ± 2.20	9.63 ± 2.26	0.88 ± 0.35	11.51 ± 1.36

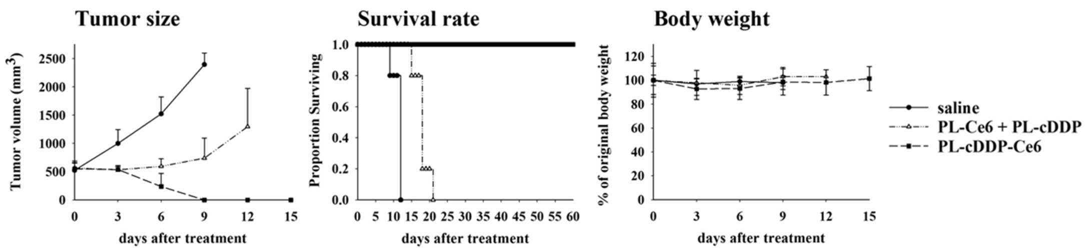

Figure S3. Therapeutical efficacy of PL-cDDP-Ce6 on nu/nu nude mice bearing H460 tumors with average tumor size of 500 mm^3 before drug administration. After liposomal drug (Ce6: 1.75 mg/kg and cDDP: 6.34 mg/kg) injection, light irradiation (100 J/cm^2) was applied onto the tumor at 2 h and 12 h, respectively. Left panel, tumor size; Middle panel, survival rate; Right panel, body weight. The

pharmaceutics

results are represented as the mean ± S.D. for each group (N = 5). Statistical analysis was performed by two-way ANOVA. Complete tumor regression was observed in all mice treated with PL-cDDP-Ce6, resulting in apparent cure with clear superiority over the treatment of PL-cDDP and PL-Ce6 in combination.
